# Borderline personality disorder and adolescent suicide attempt: the mediating role of emotional dysregulation

**DOI:** 10.1186/s12888-021-03377-x

**Published:** 2021-08-09

**Authors:** Bojan Mirkovic, Véronique Delvenne, Marion Robin, Alexandra Pham-Scottez, Maurice Corcos, Mario Speranza

**Affiliations:** 1 Université Paris-Saclay, UVSQ, Inserm U1018, CESP, “DevPsy”, 94807 Villejuif, France; 2 Pôle de psychiatrie de l’enfant et de l’adolescent, Nouvel Hôpital de Navarre, Université de Normandie, Nouvel Hôpital de Navarre, route de Conches, 27000 Évreux, France; 3 Service de Pédopsychiatrie, Hôpital Universitaire des Enfants Reine Fabiola, Brussels, Belgium; 4 Département de Psychiatrie de l’Adolescent et du Jeune Adulte, Institut Mutualiste Montsouris, Paris, France; 5 GHT Paris Psychiatrie et Neurosciences, Centre Hospitalier Sainte Anne, Paris, France; 6 Service Universitaire de Psychiatrie de l’Enfant et de l’Adolescent, Centre Hospitalier de Versailles, Versailles, France

**Keywords:** Emotion regulation, Adolescent, Suicide attempt, Suicidal behaviours, Borderline personality disorder

## Abstract

**Background:**

Emotional dysregulation seems to be a core feature of Borderline Personality Disorders (BPD). In addition, recent research in the adolescent population has shown that suicidal behaviours have been associated with maladaptive strategies of emotion regulation.

**Methods:**

This study examined the relative contributions of emotional dysregulation to suicide attempt history in a clinical sample of borderline adolescents. Data were analyzed from 85 participants of the Collaborative European Research Network on Borderline Personality Disorder. Participants completed measures of BPD traits and symptoms, suicide behaviours, emotional dysregulation, attachment styles and lifetime depressive disorders.

**Results:**

In an SEM model, lifetime depressive disorders and insecure attachment styles have a significant direct effect on lifetime suicide attempt, but only lifetime depressive disorders have an indirect effect through emotion dysregulation. The results suggest that emotional dysregulation has a mediating role in suicide attempts among BPD adolescents.

**Conclusions:**

These findings call for the development of interventions targeting the role of emotion dysregulation in effectively predicting and preventing suicidality in borderline adolescents.

## Background

Borderline Personality Disorder (BPD) in adolescents is now recognized as a legitimate disease [1] deserving early intervention and treatment [2, 3]. BPD has been found in 1.6 to 3% of the general population [1, 4]. In mental health institutions, BPD among adolescents is common, with an estimated prevalence of 10% in ambulatory patients [5], nearly 50% in hospital care units [6, 7] and more than 80% in cohorts of suicidal adolescents [8]. BPD is strongly associated with suicidal behaviours (SB) [9–11], this risk increases during the course of the disorder [11], in the presence of psychiatric comorbidities [12] and predicts significant functional impairment across a number of domains [13]. The severity of the disorder is such that up to 10% of people with BPD die by suicide [14, 15]. To date, the development of BPD is best explained by a biosocial model [16, 17]. This model suggests that emotion dysregulation is a central BPD feature that leads to the behavioral dysregulation observed in the disorder. Although the mechanisms underlying the relationship between suicidality and emotion dysregulation are not entirely understood, Linehan’s theory [17] suggests that subjects who have attempted suicide experience high levels of emotional distress following difficult events that they consider to be aversive and intolerable and cannot generate and implement adaptive solutions.

Emotion dysregulation is the inability to respond flexibly to, and control, emotions [18]. There is considerable variation in the phenomena studied under the heading of ‘emotional dysregulation’ in BPD, including emotion sensitivity, heightened and labile negative affect, a deficit of appropriate regulation strategies, and a surplus of maladaptive regulation strategies [19]. One way to understand these disparate approaches is to view emotional dysregulation not as an end state, but as a process incorporating multiple interactive components [20]. In the clinical population of adults with BPD, numerous studies have clearly identified difficulties with emotion regulation as being associated with SB [21–24]. Moreover, even in non-clinical populations, emotional regulation difficulties have been shown to be correlated with suicidality [25, 26]. BPD features have prospectively predicted SB even when the non-suicidal self-injury criterion of BPD was excluded, indicating that the association with BPD features is not simply due to criterion overlap [23]. However, Yen et al. [23] showed that only the affective instability feature prospectively predicted suicide attempts (SAs) even when controlling for negative mood states such as major depression. In addition, emotional dysregulation seems to be involved in several risk factors associated with SB such as self-injury [27, 28], impulsivity [29], substance abuse [6], depressive disorders [30] or interpersonal difficulties [31]. However, studies in adolescent populations remain rare despite the high prevalence of BPD in suicidal adolescents [8]. In a previous study with adolescents, suicide attempters reported greater affect dysregulation than suicide ideators [32]. Moreover, according to Glenn et al. [33], the affective instability facet of adolescent BPD was related to SA even when controlling for general negative emotionality. However, in a sample of high-risk suicidal adolescents, Yen et al. [11] found no difference in emotional regulation between BPDs and non-BPDs. On the other hand, the authors found that those with more BPD criteria had higher levels of negative affectivity, depression, anxiety and higher affective lability. In summary, the conflicting results reported in studies examining emotional dysregulation and SB suggest the notion that the nature of the relationship depends on the presence or absence of other variables such as mood depressive disorders (MDD) or interpersonal difficulties. Thereby about 65% of youths and adults with BPD have MDD in their lifetime [34]. Links between MDD and BPD are sometimes strong and the clinical distinction between them is not clear, especially in adolescents [7, 34, 35]. Adult studies have shown that while there is significant overlap or co-morbidity between BPD and DD [12, 24], these are probably distinct clinical phenotypes.

SBs are often related to emotions connected with interpersonal relationships [31, 36, 37]. For example, almost 40% of adolescents indicated that friendship difficulties were the main precipitant to their suicide attempts [36]. Furthermore, the quality of interpersonal relationships is a key factor in the psychopathology of borderline adolescents, potentially linked to insecure or disorganized attachment patterns [38, 39]. The quality of attachment is defined in particular by the balance the subject achieves between knowing when to seek help from particular figures (figures of attachment) in cases of distress and relying on their own resources to overcome a challenge or crisis [40]. The feeling of helplessness and hopelessness generated by interpersonal difficulties are important predictors of SB [41]. In addition, it has been suggested that interpersonal and experiential disturbances that are features of BPD are associated with the traits of affective instability among patients with personality disorders [42].

In summary, despite the growing evidence in adults, much less is known about the relations between emotional dysregulation and SB in BPD adolescents. Based on previous research, the present study aims to better understand the mechanisms underlying suicide attempts in BPD adolescents. Specifically, we seek to clarify the role of emotional dysregulation in suicide attempt in adolescents with BPD according to the presence/absence of a MDD and/or relationship difficulties. We hypothesize that the relationship between suicide attempt, MDD and relational difficulties is partially mediated by emotional dysregulation.

## Methods

### Participants

The study sample was taken from a European research project on borderline disorder in adolescence (European Research Network on Borderline Personality Disorder, EURNET BPD; for a full description of the study’s methodology, see [43]). The research network was composed of 5 academic psychiatric centers in France, Belgium, and Switzerland. During the period from January to December 2007, all in- and out-patient adolescents (15 to 19 years old) were clinically screened by the consulting psychiatrists following the DSM-IV criteria for borderline personality disorders. Adolescents fulfilling a clinical diagnosis of BPD were referred to the research team for further assessment as described below. People suffering from schizophrenia or any potentially serious medical condition were excluded. 107 patients with a possible diagnosis of BPD were referred to the study by their clinicians. Of these subjects, 85 fulfilled SIDP-IV criteria for a BPD.

### Procedure

Axis-II disorders were ascertained through the Structured Interview for DSM-IV Personality (SIDP-IV), a semi-structured interview for assessing each of the ten DSM-IV personality disorders, including BPD [44]. Axis-I disorders were assessed using the Schedule for Affective Disorders and Schizophrenia for School-Age Children (K-SADS) which is a semi-structured interview for the assessment of both past and current psychiatric disorders in children and adolescents [45]. Diagnostic interviews were conducted by a research team of 5 doctoral or master’s level clinicians (psychologists or psychiatrists) who were familiar with DSM-IV Axis-I/II disorders and were experienced in the assessment and/or treatment of psychiatric adolescents. Final research diagnoses were established by the best-estimate method on the basis of the interviews and any relevant additional data from the clinical record, according to the LEAD standard [46]. Inter-rater reliability for SIDP-IV was calculated from independent ratings of ten videotaped interviews. The Kappa coefficient for the presence / absence of a BPD was very good (0.84). The intraclass correlation coefficient for borderline SIDP-IV score was excellent (0.95). At the end of the assessment session, an overall level of psychosocial functioning was calculated for each patient according to the Global Assessment of Functioning (GAF) [47].

### Measures

#### Assessment of emotional dysregulation

Emotion regulation is itself a complex trait and several psychometric tools have been developed over time. For the present study we created *a composite emotional dysregulation score* from three scales investigating emotional processes:

(i) The Affective Lability Scale (ALS) [48] measures emotional statesin 3 dimensions: perceptions of changes in emotion and associated cognition, perceptions of physiologic changes, and perceptions of behavioral changes. It is a self-questionnaire examining the variation between the euthymic state and four affective states: depression, elation, anger and anxiety, as well as the variation between elation and depression, and between anxiety and depression. An English and French version for adolescents was developed by Guilé et al. [49]. Internal consistency is high for each section and for the entire instrument (Cronbach α = 0,87 to α = 0,95). In an adult BPD sample, Yen et al. [23] showed that affective lability was associated with SB and was the only significant predictor of future suicide attempts;
(ii) The Bermond and Vorst Alexithymia Questionnaire (BVAQ) [50] has a 5-factor structure: B1: ability to verbalize; B2: ability to fantasize; B3: ability to introspect; B4: emotional reactivity; B5: ability to analyze (Cronbach α = 0,85) [51]. Alexithymia may also be considered as a deficit in the treatment and regulation of emotions [52]. It has been shown that alexithymic adolescents have more psychiatric disorders such as depression [53] or BPD [54]. In addition, alexithymia appears to be a risk factor for suicidal behaviours [55]. For the composite score of the study, we only included the Affective score of the BVAQ (B2 + B4 factor scores).
(iii) The Cyclothymic-hypersensitive temperament questionnaire (CHTQ) is a child and adolescent French modification of Akiskal’s cyclothymic temperament questionnaire (Cronbach α = 0,88) [56, 57]. The cyclothymic temperament is characterized by permanent instability of mood, thinking and behaviors [58], which is frequent in bipolar disorders [59]. Several studies have shown that cyclothymic temperament is associated with SB in adults [60, 61], in adolescents with mood disorders [62], and also in community populations [63].

The composite score from these three scales allows a broader evaluation of the notion of emotional dysregulation in the sense of emotional and affective variability. Indeed, the notion of emotional variability may refer either to the ways of dealing with an emotional situation, or to the notion of emotional vulnerability (the variables relating to the experience or the quality of the emotion), or to the ability to understand emotions.

#### Definition of suicide attempt

The event qualified as an attempted suicide if it met the Silverman definition. According to Silverman et al. [64], suicide attempt is defined as self-inflicted, potentially damaging behaviour whose outcome is not fatal and for which there is evidence of intent to die. The lifetime occurrence and number of suicide attempts were directly assessed during a clinical interview with the adolescent and their parents or guardians.

#### Assessments of interpersonal relationships

The Relationship Scales Questionnaire (RSQ) [65] is a combined measure of attachment. Attachment style is a comprehensive framework for understanding how emotional bonds and relationships develop [66] and could not be considered synonymous with interpersonal difficulties. The RSQ is a 30-item continuous-scored scale with four subscales that measure the degree to which individuals exhibit four attachment patterns: secure, fearful, dismissing, and preoccupied. A “secure” attachment style (positive models of self and others) indicates a sense of one’s own value and importance to others, as well as a belief that others are generally available and helpful in times of need, and that they accept us as we are. The “preoccupied” style (negative self-model and positive model of others), is characterized by the feeling that we have little value for others and at the same time, that we expect positive responses from them while fearing that they are not as reassuring and comforting as we would like. The “detached” style (positive model of oneself and negative model of others) implies a feeling of one’s own value that one owes only to oneself and very negative expectations of others from whom, on the other hand, one should expect nothing. Finally, the “fearful” style (negative models of self and others) combines a feeling of personal non-value in the eyes of others and the belief that others are neither available nor benevolent when needed. In a French validation study Cronbach’s coefficient was low for the prototypical scales (0.41 for “secure”, 0.54 for “fearful”, 0.22 for “preoccupied”, and moderate for “dismissive” (0.64) [67]. Zortea et al. conducted an extensive psychometric study of the RSQ in a sample of adults and suggested that a two-dimensional (i.e., anxiety and avoidance) approach to assessing adult attachment was optimal.

#### Others assessments

The Beck Depression Inventory (BDI) [68] is a well-known instrument for assessing the intensity of depression in clinical and normal patients (Cronbach α = 0.86 for psychiatric patients and 0.81 for nonpsychiatric subjects). Borderline disorder was investigated using the Revised Diagnostic Interview for Borderlines (DIB-r) [69]. The DIB-R is a semistructured interview (Cronbach α = 0.86) comprising 105 items and 22 summary statements for assessing the persistence of symptoms of BPD over the course of the past two years and offers a more comprehensive characterization of BPD, such as affective, behavioural, interpersonal, and cognitive phenotypes [70].

To determine the severity of BPD symptoms, the participants completed the McLean Screening Instrument for BPD, Cronbach α = 0.87 [0.84;0.90] (MSI-BPD; [71, 72].

#### Data analytic plan

First, variables of interest were described. The parameters chosen for quantitative variables were mean and standard deviation. The parameters chosen for qualitative variables were number of occurrences and frequency. We then ran univariate comparison of variables of interest between two groups, those who had zero lifetime SAs versus those who had at least one lifetime SA. Depending on the validity of the assumptions, quantitative variables were either tested using Welch t-test or Wilcoxon rank sum test. Qualitative variables were either tested using Chi-square test without continuity correction or Fisher’s exact test. Secondly, the mediating role of emotional dysregulation on the relationship between SA, DD and relational difficulties was assessed within the structural equation modelling framework (SEM) with R lavaan package. This method is called path analysis, which is a special case of SEM when there are no latent variables in the model. Model specification was done using prior knowledge. Outcome was the number of lifetime suicide attempts, covariates were any moods disorders and RSQ and the mediator was emotional dysregulation. Concerning the RSQ we have excluded the items contributing to the secure attachment style. We present results from the SEM, including covariates’ direct effects and indirect effects through mediation by emotional dysregulation. Raw and standardized maximum likelihood estimates are presented. Since the multinormality assumption was not met, bootstrapped *p*-values (R = 10,000 replications) were chosen for inference. Sensitivity analyses were run using robust estimators. Analyses were performed with R 3.5.1 software. A p-value under 0.05 was considered significant.

## Results

### Descriptive statistics and group differences

Of the initial cohort, variables of interest were fully available for 75 adolescents (11 boys, 15%, 64 girls, 85%). The mean age was 16.3 years (SD = 1.4) and 67% (*N* = 50) were inpatients. Of the 75 participants, 15 (20%) had not attempted suicide, 23 (31%) had committed one SA and 37 (49%) had committed two or more SAs. Of those who had attempted suicide, the lifetime average was 1.72 (SD = .45) The main modes of attempted suicide were drug poisoning (75%), strangulation (9%) vein cutting (8%) and jumping (4%). Table 1 shows the scores for all the variables of interest and the comparisons between the participants who had not attempted suicide and those with at least one SA. Univariate analysis yielded several significant differences for the Beck Depression Inventory, all of the DIB-R subscales (affect, cognition, impulsive actions and interpersonal relationships) and the severity of BPD symptoms (MSI-BPD). Also, there was no difference in terms of sex and age, cyclothymic-hypersensitive temperament questionnaire (CHTQ) and non-secure attachment style (RSQ). The participants who had not attempted suicide show better overall functioning, as assessed by their GAF score: mean (SD) 56.42 (10.19) vs. 41.05 (16.61), *p* = .03. Lifetime axis-I disorders according to the presence of suicidal attempts are presented in Table 2. Axis-I diagnoses were included as groups of related disorders.

**Table 1:** Descriptive information for each main study variable and comparisons by suicide attempt status

	SA = 0		SA ≥ 1		p
	*n* = 15		*n* = 60		
	*Mean*	*SD*	*Mean*	*SD*	
**Age**	16.47	1.6	16.62	1.46	.91
**Depression scale (BDI-II)**	25.64	11.22	32.64	13.54	**.04**
**Attachment style (RSQ)**					
Secure	17.20	3.90	13.8	3.98	.09
Fearful	12.67	4.33	12.85	3.56	.96
Preoccupied	12.9	2.19	14.05	3.09	.17
Dismissing	16.22	4.29	13.41	4.68	.12
**DIB-R**					
Affects	0.791	0.220	0.907	0.170	**.01**
Cognition	0.308	0.261	0.500	0.325	**.007**
Impulsive actions	0.585	0.212	0.721	0.223	**.009**
Interpersonal relationships	0.396	1.80	0.524	0.199	**.006**
Global score	6.469	1.739	8.026	1.739	**.0005**
**MSI-BPD**	6.25	2.22	7.89	2.43	**.029**
**Affective Lability Scale**	7.07	3.88	10.01	2.85	.06
**BVAQ Affective score**	23.4	2.8	27.8	3.09	.08
**BVAQ Cognitive score**	35.4	3.57	35.18	4.07	.85
**CHT Questionnaire**	15.75	5.31	17.5	4.28	.34
**Female sex, N (%)**	12	80	53	88.3	.40

*SA* Suicide Attempt, *RSQ* Relationship Scales Questionnaire, *DIB-R* Revised Diagnostic Interview for Borderlines, *MSI* McLean Screening Instrument, *BVAQ* Bermond and Vorst Alexithymia Questionnaire, *CHT* Cyclothymic-hypersensitive temperament

**Table 2:** Axis I lifetime comorbidity according to the presence or absence of suicidal attemps

	SA = 0	SA ≥ 1	p
	*n* = 15	*n* = 60	
	%	%	
**Axis I – Lifetime diagnoses**			
Major depression	13.3	66.7	**< .001**
Any Depressive Disorders	33	73.1	**.004**
Anorexia nervosa	15.2	35.0	.17
Bulimia nervosa	18.2	14.3	.12
ADHD	18.2	16.7	.75
Conduct disorder	18.2	23.8	1.0
Oppositional disorder	21.2	24.3	1.0
Any substance related disorders	13.2	23.2	.39

*SA* Suicide attempt, *ADHD* Attention deficit hyperactivity disorder

### Structural equation model

**Fig. 1:**
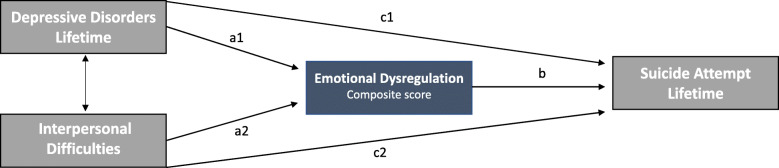
An illustration of the proposed SEM model of depressive disorders and interpersonal difficulties influencing suicide attempt as mediated by emotion dysregulation

**Fig. 2:**
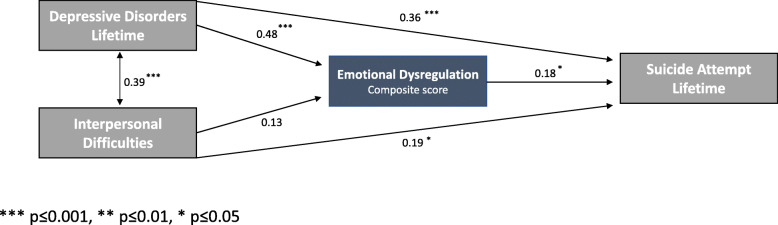
Final SEM model of depressive disorders and interpersonal difficulties influencing suicide attempt as mediated by emotion dysregulation

The hypothesized model is illustrated in Fig. 1. Path a1 represents the effect of the independent variable (MDD current and past) on the mediator (emotional dysregulation), and path b the effect of the mediator on the dependent variable (lifetime SA). Path c1 is the direct effect of lifetime MDD on lifetime SA. The same model applies to the second variable: insecure attachment. Path a2 represents the effect of insecure attachment on the mediator, and path c2 the direct effect of insecure attachment on lifetime SA. Results of the mediation analyses to test the significance of the direct and indirect effects of lifetime MDD and insecure attachment on SA through emotion dysregulation are displayed in Table 3 and Fig. 2**.** As expected from model specification, the model is saturated and the fit cannot be assessed (CFI = 1 and RMSEA = 0).

**Table 3:** Summary of multiple mediation analysis for emotion dysregulation and suicide attempt lifetime

	Estimate	Std.Err	z-value	*p*-value	Std.All
**Regressions: Suicide Attempt Lifetime**					
Emotional Dysregulation (b)	.114	.052	2.205	**.02**	.184
Depressive disorders Lifetime (c1)	.938	.225	4.166	**.0001**	.361
Interpersonal Difficulties (c2)	.130	.056	2.336	**.019**	.195
**Regressions Emotional Dysregulation**					
Depressive disorders Lifetime (a1)	2.001	.365	5.478	**.0001**	.481
Interpersonal Difficulties (a2)	.138	.092	1.499	.134	.130
**Covariances: MDD Lifetime**					
Interpersonal Difficulties	.376	.081	4.628	**.001**	.387

*MDD* Mood Depressive Disorders, (*N* = 75; 10,000 bootstraps)

*Std.All* Both latent and observed variables are standardized

Lifetime MDD has a significant direct effect on the dependent variable (path c1: Estimate = .938, Std. Err = .228, Std.all = .361, *p* < .001) and an indirect effect through emotion dysregulation (path a1b: Estimate = .228, Std. Err = .109, Std.all = .088, *p* = .036). In addition, we found a significant overall effect (paths a1b + c1: Estimate = 1.165, Std. Err = .196, Std.all = .449, *p* < .001) on SA lifetime.

Insecure attachment style has a significant direct effect (path c2: Estimate = .130, Std. Err = .053, Std.all = .195, *p* = .019) on the dependent variable. We did not find a significant indirect effect through emotion dysregulation on SA lifetime (path a2b: Estimate = .016, Std. Err = .013, Std.all = .024, *p* = .219). However, the overall effect (a2b + c2) is significant (Estimate = .146, Std. Err = .056, Std.all = .218, *p* = .009).

## Discussion

In this study we sought to examine the contribution of emotional dysregulation to SA in borderline adolescents. We proposed a SEM model of lifetime SA from 85 participants of the Collaborative European Research Network on Borderline Personality Disorder, a high-risk sample of adolescents with BPD. We predicted that (i) lifetime MDD and interpersonal difficulties would be positively associated with the number of lifetimes SAs; (ii) that the association between lifetime MDD and SAs would be partially related to emotional dysregulation; (iii) and lastly that the association between interpersonal difficulties and SA would be partially related to emotional dysregulation.

Concerning our SEM model, two results may be highlighted. Firstly, the mediational results point to the direct effects of MDD on lifetime SAs and their indirect effects through increasing emotional dysregulation and indicate that the overall effect may be an important risk factor for SA in borderline adolescents. Secondly, we have shown that relational difficulties, specifically insecure attachment, had a direct effect on lifetime SAs but had no indirect effect through emotional dysregulation. This last point deserves to be underlined and discussed. Interpersonal conflicts have been identified as frequent precipitating factors in the adolescent population. In addition, interpersonal difficulties, linked to attachment disorders, represent a central element of borderline functioning. More specifically, traumatic attachments during the first years of life appear either to be strongly associated with the genesis of well-defined mental disorders (e.g. traumatic-dissociative disorders) or to occur variably in many other diagnostic categories, a fact which complicates their clinical picture and worsens their prognosis [73]. Manifestations of dissociation are very common in people with trauma-related disorders such as PTSD or BPD [74], and are supposed to be generated by the disintegrative processes activated by the traumatic experience [75].

Borderline participants who have more difficulty in regulating their emotional experiences are at greater risk of SA. These results are consistent with research that has demonstrated that BPD-related emotional dysregulation predicts a range of prospective negative outcomes, including SA [23, 76]. Yen et al. [23] showed in a prospective 2-year study of a cohort of 621 adult borderline subjects that only the affective instability feature of BPD predicted SA. Similarly, Glenn et al. [33] showed similar results in adolescent populations. In a sample of 97 borderline adolescents the authors found that the affective instability facet of BPD was uniquely related to SB and uniquely differentiated adolescent ideators and attempters.

As expected, our multivariate analyses also showed very strongly that MDD is a major risk factor for SA in borderline adolescents. This is consistent with the literature data that show that major depressive disorders are the leading cause of suicide with a contribution of more than 60% [9] and a 40 times greater risk of completing a suicide [77].

Participants who had attempted suicide did not have higher scores on the scales for assessing emotional dysregulation; however, it should be noted that the scores were generally high in both groups. Two hypotheses may explain this result. On the one hand, the fact that both groups comprised BPD subjects and that emotional dysregulation is a central feature of this disorder [17, 23, 24] produces a limited range of variance that precludes any differentiation between the two groups. From a neurodevelopmental point of view, it is interesting to note that at this age there are a set of neurobehavioural changes that significantly influence the motivation and regulation of emotions [78]. Several models of adolescent brain development have suggested that a maturation gap between cognitive control and emotional processes may explain why adolescents tend to make more emotional decisions, which may lead to increased risk behaviours [79, 80]. These models emphasize the faster maturation of the sub-cortical affective areas compared to the slower-maturing frontal cortical areas. However, as Crone and Dahl [81] have pointed out, there are also specific social and emotional changes that occur during puberty due to the subject’s environment and not simply due to developmental immaturity.

There are some limitations to our study, which invite to cautious interpretations of the findings and their generalizability. Firstly, the use of a cross-sectional design limits the conclusions that can be drawn about the role of interpersonal difficulties, depressive disorders and emotional dysregulation in the development and maintenance of SB. Although structural equation modelling did not support an alternative direction of effects, longitudinal modelling is needed to clarify these questions. Secondly, the self-assessment methodology used may be subject to a registration bias, such as that deriving from social desirability. A third limitation of this research concerns the generalization of the results. Although the entire sample was derived from 5 recruitment centers in 3 European countries, the responses may not be transferable to other populations, mainly because of the high proportion of hospitalized patients. Thus, a Berkson-type selection bias is possible. Finally, questions may be asked as to the validity of the composite score for emotional dysregulation. There are several justifications for creating a composite for emotional dysregulation. Firstly, the notion of emotional dysregulation is broad and is thus difficult to evaluate it using a single psychometric tool. Secondly, the notion of emotional variability may refer either to the ways of dealing with an emotional situation, or to the notion of emotional vulnerability (the variables relating to the experience or the quality of the emotion), or to the ability to understand emotions. Thirdly, even if the three selected scales do not share a common construct, all three reflect a conceptual entity. Moreover, we found it appropriate to include a degree of affective alexithymia. Alexithymia is defined as poor awareness of emotions and a diminished ability to think and talk about feelings [52]. Several authors have suggested links between alexithymia and emotional dysregulation [82]. Indeed, people who are unaware of their feelings have greater difficulty modulating their emotional excitement, especially when they perceive a potential danger in their relational environment. In this case, the relational conflict may be simply processed as tension or distress, thus increasing the risk of suicidal behaviour [83, 85]. 

This study has also several strengths. Firstly, the presence of a consistent structured diagnostic procedure for all the participants in the clinical sample for Axis I and Axis II (LEAD Standard). Moreover, all the adolescents for whom there was no consensus between investigators for the diagnosis of BPD were excluded. Secondly, we considered SA only if the suicidal intention was clearly identified.

The results of our study could be considered in the light of the Alternative DSM-5 Model for Personality Disorders who considers personality disorders as characterized by impairments in personality functioning associated with specific pathological personality traits [84]. Disturbances in self and interpersonal functioning constitute the core of personality psychopathology and are evaluated on a continuum. Dimensional models of BPD could be particularly pertinent in adolescents, as a dimensional approach may better account for the developmental variability and heterogeneity observed during this age period. The current literature suggests that the central dysfunctional domains of BPD are affective dysregulation, separation insecurity, depressivity, impulsivity and risk-taking, which are intrinsically linked to each other and possibly linked to an insecure attachment style [85].

Overall, the findings of the present study suggest that emotional dysregulation may be a significant risk factor for SA in adolescents with BPD. Our results support suicide prevention efforts with adolescents that target emotional dysregulation. And in fact, several studies have shown the effectiveness of DBT-A programs specifically targeting emotional dysregulation in reducing suicidal behaviour in adolescents [86–88]. Other approaches targeting interpersonal difficulties and emotional dysregulation such as mentalization therapy have also shown encouraging results. For example, the RCT conducted by Rossouw et al. [89], showed that mentalization-based therapy reduced the recurrence of TS at 12 months in adolescents with multiple self-harm or borderline personality disorders.

## Conclusions

The results of our study suggest that emotional dysregulation should be considered as a primary target of psychotherapeutic approaches of borderline adolescents as a strategy to improve the handling of interpersonal difficulties and potentially prevent suicidal behaviours. However, further longitudinal studies are required to examine how affective instability can predict future suicidal behaviors in borderline adolescents over time and in relation to comorbidities and the social environment.

## Data Availability

The datasets used and analysed during the current study are available from the corresponding author on reasonable request.

## Abbreviations

BPD: Borderline Personality Disorder
SA: Suicide Attempt
MDD: Mood Depressive Disorders
MSI-BPD: McLean Screening Instrument for Borderline Personality Disorder
SIDP-IV: Structured Interview for DSM-IV Personality
DIPD-IV: Diagnostic Interview for DSM-IV Personality Disorders
